# Hypertrophic Versus Atrophic Photoaging in Monozygotic Twins: Insights from a Unique Vitiligo Case Report

**DOI:** 10.3390/diagnostics16121768

**Published:** 2026-06-08

**Authors:** Ilaria Proietti, Vincenzo Coppolelli, Alberto Taliano, Annunziata Dattola, Steven Paul Nisticò, Giovanni Pellacani, Concetta Potenza, Stefania Guida

**Affiliations:** 1 Dermatology Unit “Daniele Innocenzi”, “A. Fiorini” Hospital, 04019 Terracina, Italy; ilaria.proietti@uniroma1.it (I.P.); concetta.potenza@uniroma1.it (C.P.); 2 Dermatology Unit, Department of Clinical Internal, Anesthesiological and Cardiovascular Science, University of La Sapienza, 00161 Rome, Italy; albertotaliano1@gmail.com (A.T.); annunziata.dattola@uniroma1.it (A.D.); steven.nistico@uniroma1.it (S.P.N.); pellacani.giovanni@uniroma1.it (G.P.); 3 Unit of Dermatology, IRCCS Ospedale San Raffaele, 20132 Milano, Italy; guida.stefania@hsr.it

**Keywords:** photoaging, vitiligo, UV exposure, monozygotic twins

## Abstract

This report describes a case of 62-year-old monozygotic twin sisters showing markedly different patterns of cutaneous aging despite identical genetics. One twin, affected by long-standing non-segmental vitiligo, exhibited features of hypertrophic photoaging, including pronounced wrinkling and dermal thickening, while the unaffected twin showed an atrophic phenotype with smoother, thinner skin. Imaging findings supported these differences. The case highlights the complex interplay between ultraviolet exposure, genetic factors such as MC1R, and non-genetic influences, suggesting that melanocytic loss and environmental factors contribute significantly to divergent skin aging outcomes.

**Figure 1:**
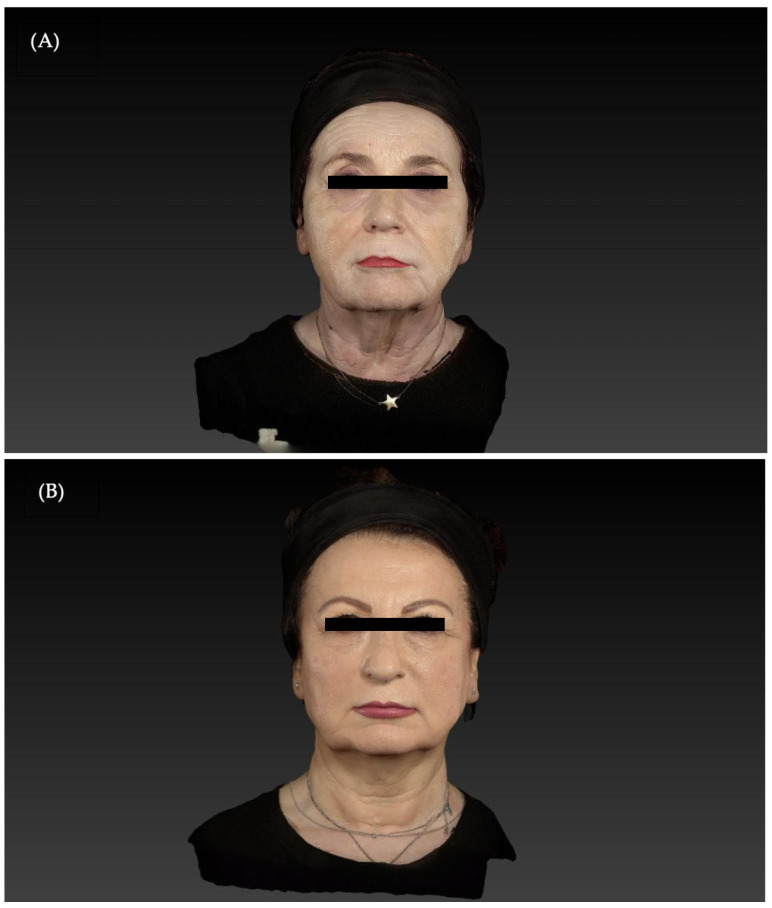
Clinical comparison between 62-year-old monozygotic twin sisters demonstrating distinctly divergent cutaneous aging phenotypes despite identical genetic backgrounds. One twin has longstanding progressive non-segmental vitiligo, whereas the other remains unaffected. They came to our attention during a dermatological consultation they attended to explore recent advancements in vitiligo therapies. Neither twin has a history of chronic medical conditions or regular medication use. Both are lifelong non-smokers and work predominantly indoor, with one as an accountant and the other one as a school administrator. Similar sun-protection behaviors were reported, consisting of the application of SPF 50+ sunscreen exclusively during the summer season. On clinical examination, the twin with vitiligo exhibited fine rhytides, dermal laxity, and a coarse, leathery texture—consistent with the hypertrophic photoaging (HP) phenotype—particularly affecting depigmented areas in the perioral, zygomatic, and frontal regions (**A**). In contrast, the unaffected twin displayed smoother, more translucent skin with decreased dermal thickness and less pronounced wrinkling, characteristic of the atrophic photoaging (AP) phenotype (**B**). While UV exposure is the principal environmental factor, the response to photodamage is modulated by intrinsic variables such as sex, hormonal milieu, dermal vascular density, and genetic predisposition [1]. Among the molecular determinants, the melanocortin 1 receptor (MC1R) gene has earned particular interest due to its role in melanogenesis and UV response: MC1R is a highly polymorphic gene that encodes a receptor located on the cell membrane, primarily involved in the regulation of melanogenesis through its interaction with α-melanocyte-stimulating hormone (α-MSH). Numerous genetic variants of MC1R have been identified, with several of them affecting the receptor’s ability to promote normal pigment production, such as the one associated with red-haired color phenotype. Research has also highlighted additional biological roles of MC1R, including protection against oxidative stress and participation in DNA damage repair pathways: as a result, alterations in this gene have also been associated with an increased predisposition to photoinduced skin damage and the development of cutaneous malignancies. Polymorphic variants of MC1R, especially those impairing receptor function, have been associated with the HP phenotype, presumably due to reduced melanin-mediated photoprotection [2,3]. Moreover, MC1R is also expressed—albeit at lower levels—on dermal fibroblasts, where its functional significance remains unclear; in addition, in vitro studies have demonstrated that human skin fibroblasts are capable of producing proopiomelanocortin-derived peptides, including adrenocorticotropin, α-MSH and b-endorphin. While its role in these cells is likely limited, MC1R signaling may contribute indirectly to cutaneous homeostasis through modulation of inflammatory pathways or fibroblast-derived paracrine signals. In particular, fibroblasts are known to influence pigmentary regulation by secreting mediators such as TGF-β, which inhibits MITF expression and suppresses melanocyte activity [4,5].

**Figure 2:**
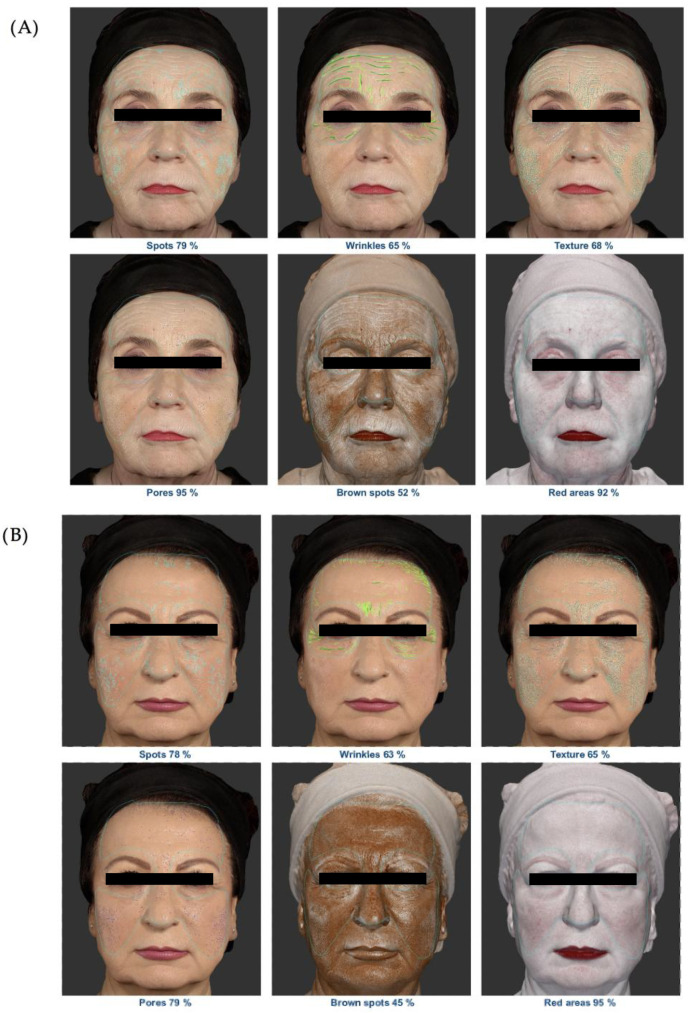
Complementary 3d skin analysis (obtained with VECTRA H2 3D Face Sculptor–Canfield) was performed: this technology, through stereophotogrammetry and high-resolution imaging, combines multiple photographs taken from different angles to generate a three-dimensional facial model. The software then maps skin texture and color onto the 3D surface, enabling quantitative assessment of facial morphology, skin texture, wrinkles, pigmentation, and volumetric changes. This approach provides an objective and reproducible evaluation of facial features and treatment outcomes. This analysis further highlighted differences between the twins: the hypertrophic aging twin demonstrated a predominance of wrinkles (A), while the atrophic aging twin showed a greater presence of brown spots and red areas (B). These findings align with the established literature describing increased pigmentation and vascular features in AP, versus enhanced dermal remodeling and wrinkling in HP [6,7]. Cumulative ultraviolet (UV) exposure is recognized as a primary extrinsic driver of photoaging, typically manifesting in two clinically distinguishable phenotypes: HP and AP [6]. These phenotypes differ not only in gross morphology but also in vascular patterns, pigmentation distribution, and dermal architecture, as demonstrated by instrumental assessments, including digital imaging, multispectral analysis and optical coherence tomography [7]. Histologic and non-invasive optical studies corroborate the presence of fibrotic remodeling and altered collagen deposition in HP skin, as opposed to hypervascularization in AP [7]. The case presented here is compelling due to the coexistence, in a single individual, of vitiligo and hypertrophic photoaging—a combination suggesting that melanocytic deficiency and impaired MC1R function may predispose to excessive dermal remodeling and wrinkle formation. While previous studies have reported correlations between MC1R variants, pigmentary phenotypes, and photoaging profiles, this case underscores the complexity of gene–environment interactions in cutaneous aging. Importantly, the phenotypic divergence observed in these genetically identical individuals highlights the limitations of a purely genetic model. Epigenetic modifications, differential environmental exposures, immune-mediated melanocyte loss, and stochastic biological events likely contribute to the observed differences in skin aging. To the best of our knowledge, this is the first reported case allowing in vivo assessment of the impact of vitiligo on facial photoaging patterns in monozygotic twins, thereby offering a unique model to investigate the role of depigmentation in skin aging while minimizing genetic variability. Further research is warranted to elucidate the reciprocal interplay between melanocytes and fibroblasts in the context of photodamage, and to clarify the role of melanocortin signaling, senescence pathways, paracrine modulation and inflammatory response in the pathogenesis of distinct photoaging phenotypes.

## Data Availability

The original contributions presented in this study are included in the article. Further inquiries can be directed to the corresponding author.

## Abbreviations

The following abbreviations are used in this manuscript:

HP	Hypertrophic photoaging
AP	Atrophic photoaging
UV	Ultraviolet
α-MSH	α-melanocyte-stimulating hormone
MC1R	Melanocortin1 receptor
